# An energy-dispersive X-ray fluorescence method for analyzing Fe and Zn in common bean, maize and cowpea biofortification programs

**DOI:** 10.1007/s11104-017-3352-4

**Published:** 2017-08-01

**Authors:** Georgia E. Guild, Nicholas G. Paltridge, Meike S. Andersson, James C. R. Stangoulis

**Affiliations:** 1 College of Science & Engineering, Flinders University, GPO Box 2100, Adelaide, South Australia 5001, Australia; 2 HarvestPlus, International Center for Tropical Agriculture (CIAT), Cali, Colombia

**Keywords:** XRF, EDXRF, Biofortification, Micronutrient, Plant

## Abstract

**Background and aims:**

Biofortification breeding programs have the need for rapid and accurate screening methods to identify nutrient dense genotypes. This study explores the use of energy dispersive x-ray fluorescence (EDXRF) for the rapid screening of iron (Fe) and zinc (Zn) concentration in three coarse-grain crops; common bean, maize and cowpea.

**Methods:**

Bean, maize and cowpea seed was provided from biofortification breeding programs and analysed with Inductively Coupled Plasma - Mass Spectrometry (ICP-MS) to determine reference Fe and Zn concentrations. A subset of samples for each crop was used to calibrate for Zn and Fe and a separate set of samples used to validate the XRF method.

**Results:**

Results indicate that when analysing bean, maize and cowpea flour samples, the EDXRF results were not significantly different to the reference ICP-MS analysis, with an average difference of ± 1 mg kg^–1^ for both Fe and Zn.

**Conclusion:**

EDXRF analysis of common beans, cowpea and maize flour enables rapid and accurate analysis when screening for Fe and Zn in bean, maize and cowpea.

## Introduction

During the last decade, significant progress has been made in the international crop breeding community to boost the nutrient concentration of staple crops through a biofortification approach (Pfeiffer and McClafferty 2007a; Pfeiffer and McClafferty 2007b; Cakmak 2008). Initial efforts have focused on the development of Zn-dense wheat (*Triticum aestivum* L.) and rice (*Oryza sativa* L.), and Fedense pearl millet (*Pennisetum glaucum* L.) and common bean (*Phaseolus vulgaris* L.) (Saltzman et al. 2017). Biofortification breeding is also underway to develop Zn-dense maize (*Zea mays* L.) and cowpea (*Vigna unguiculata* L.) (Andersson et al. 2017). The aim of these breeding programs is to increase the micronutrient dietary intake without changing the diet of those targeted. It is proposed, for example, that iron-biofortified beans could provide up to 80% of the estimated average requirement (EAR) for non-pregnant, non-lactating women of reproductive age upon meeting the breeding target concentrations (Andersson et al. 2017).

Breeding programs require fast, accurate and inexpensive methods for screening large numbers of breeding lines and germplasm for nutritional traits. While a number of analytical techniques are available to screen seed samples, a common method for measuring micronutrients is inductively coupled plasma-optical emission spectrometry (ICP-OES) (Zarcinas and Cartwright 1987). Though reliable, the ICP-OES is an expensive piece of equipment, and requires highly trained analysts, expensive Ar gas of high purity, contamination free reagents and extensive sample preparation. Consequently, ICP-OES has not proven practical for many breeding programs to conduct their analysis in-house, and most samples have had to be sent away for costly analysis that is often slow to be reported.

Energy dispersive X-ray fluorescence (EDXRF) is an elemental analysis technique widely used in mining and geology sectors. The analysis is based on the principle that each element upon exposure to an x-ray of suitable energy will produce a secondary ‘fluorescent’ x-ray. The energy of this emitted X-ray is indicative of the element and the intensity is related to the concentration of that element in the sample. Consequently, it is possible to identify and quantify the elemental composition of a sample based on the X-ray spectra. The instrument is calibrated empirically with the use of calibration standards whereby the emitted X-ray intensity is correlated with the known concentration to enable quantitation of an unknown sample.

In previous studies, we investigated EDXRF as an alternative to ICP-based methods for high- throughput analysis of Zn and Fe in rice and pearl millet (Paltridge et al. 2012b), and Zn, Fe and Se in wheat (Paltridge et al. 2012a), and concluded this analytical approach is sufficiently reliable to support biofortification programs (Guild et al. 2017). Analysis was exclusively on whole grains, since grain size in these crops is sufficiently small to allow a typical EDXRF instrument to analyse the full thickness of multiple grains in a single scan. The benefits of minimal sample preparation prior to analysis (ie no acid digestion) along with rapid analysis (30–60 s per sample) has resulted in significant time and cost benefits for this analysis with less labour time (and cost) required per analysis with a minimal expense of ~US$0.15 per sample for cup preparation (Paltridge et al. 2012b).

The aim of this study was to investigate the potential of EDXRF for the analysis of Zn and Fe concentration in three coarse grained crops: common beans, cowpea and maize.

## Materials and methods

### Trial samples

Due to the lack of suitable calibration standards, a set of calibration and validation samples were developed for each of the target crops. Whole grain samples of bean were provided from the International Centre for Tropical Agriculture (CIAT) in Colombia, cowpea samples were provided from University of California, Riverside, USA and the International Crops Research Institute for the Semi-Arid Tropics (ICRISAT), India and maize samples from the International Institute of Tropical Agriculture (IITA), Nigeria. All seed samples were gamma irradiated at 50 kGray (5 Mrad) for sterilisation prior to release into Australia. Samples were ground to flour using a Retsch Mixer Mill MM 400 fitted with ZrO grinding jars and balls (Retsch GmbH & Co KG, Haan, Germany). ICP-MS analysis was conducted in duplicate at Flinders University to determine robust reference values for the seed samples. A closed-tube digestion method was used for digesting samples (Wheal et al. 2011). All samples used for the validation and calibration contained <4 mg kg^–1^ Al, indicating these samples can be considered free from soil contamination as per HarvestPlus guidelines (Pfeiffer and McClafferty 2007b; Yasmin et al. 2014).

### EDXRF

EDXRF analysis was carried out with 2 instruments: firstly, an Oxford X-Supreme 8000 with a 10-sample carousel and secondly, a Bruker S2 Ranger with a 28 capacity sample tray. Both instruments have been used in the HarvestPlus biofortification program.

Measurement conditions for Oxford Instruments EDXRF and those used for the Bruker S2 Ranger are summarised in Table 1. To ensure comparable throughput between the two instruments, the analysis time on the Bruker was only 30 s, to account for the longer time taken for sample handling with this instrument.

**Table 1:** EDXRF analysis conditions

	Oxford Instruments X-Supreme 8000		Bruker S2 Ranger
**Conditions**	**Fe**	**Zn**	**Fe & Zn**
Atmosphere	Air	Air	Air
X-ray tube	Tungsten	Tungsten	Palladium (50 W)
Voltage	26 kV	15 kV	40 kV
Current	115 µA	200 µA	240 µA
Peak Detected	Kα	Kα	Kα
Acquisition time	60 s	60 s	30 s
Tube Filter	W5	A6	Al 500 nm
Detector	SDD	SDD	SDD

*SDD* Silicon Drift Detector

Analyses were conducted in supplied sample cups prepared as reported previously (Paltridge et al. 2012a, b; Guild and Stangoulis 2016), with 4 μm Poly-4 XRF sample film used to seal one end of the cup. Sample cups were cleaned and prepared prior to each analysis to minimise cross-contamination between samples. Samples of >5 g were used for all analysis to ensure samples were “infinitely thick” in terms of EDXRF analysis (Paltridge et al. 2012b).

### Glass standards

10 custom-made 40 mm diameter glass disks (FLUXANA® GmbH & Co. KG Borschelstr. 3, 47,551 Bedburg-Hau, Germany) with a range of nominal Fe and Zn levels were used to establish a non-matrix matched calibration for each of the crops tested (Guild and Stangoulis 2016).

### Statistics

Statistical calculations are defined below as per the literature (Perring and Andrey 2003).

**Table:** 

Concentration determined by ICP-MS	*y_i_*
Concentration determined by EDXRF	y^i
Bias	∑i=1n(y^i−yi)n
Standard error of prediction (SEP)	∑i=1n(y^i−yi)2n
Standard error of calibration (SEC)	∑i=1n(y^i−yi)2n−p−1
Limit of Quantification (LOQ)	$\frac{N[\text{analyte}]}{S}10$

## Results

### Calibration

**Fig. 1:**
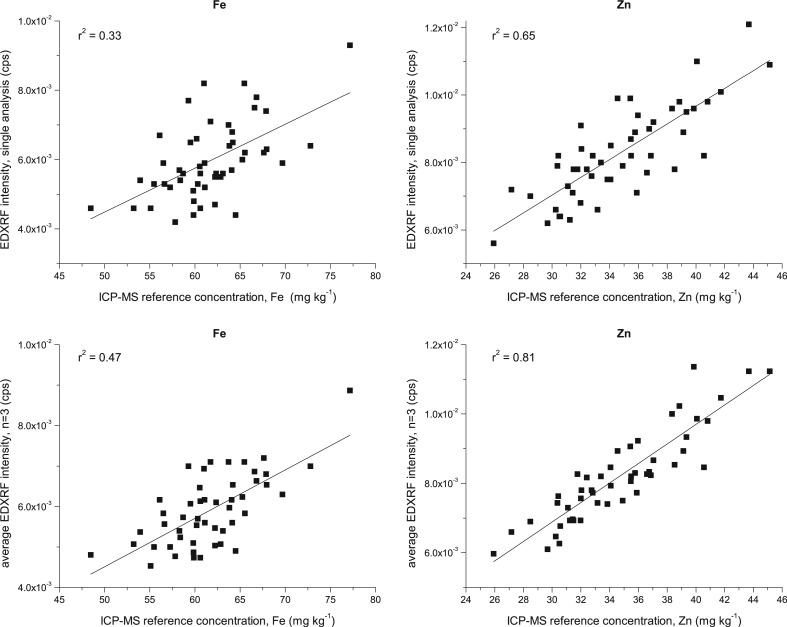
Correlation between EDXRF intensity and ICP-MS reference concentration for Fe and Zn in 50 whole grain cowpea samples with (top) single scan analysis and (bottom) averaged intensity for triplicate EDXRF scans for Fe and Zn (left and right respectively)

Preliminary analysis of cowpea was attempted with whole grain samples; however, it was evident that the correlation between the EDXRF intensity and the ICP-MS reference value was not strong, particularly for screening Fe, with r^2^ < 0.5 even when using averaged triplicate scans (Fig. 1). Consequently, it was determined that grinding samples would be required to obtain suitable calibrations for screening these large seeded crops.

**Fig. 2:**
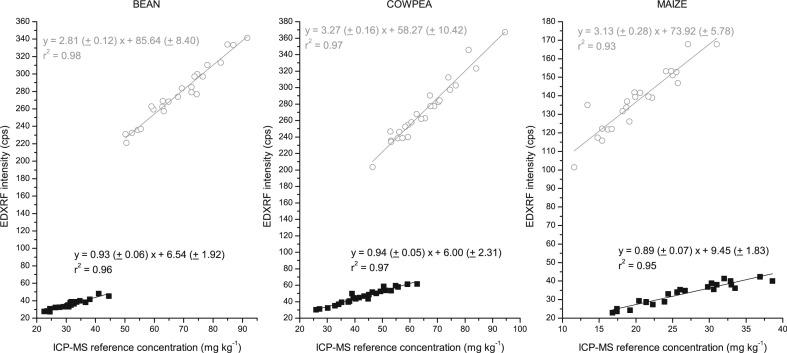
EDXRF calibration for bean, cowpea and maize flour with the Oxford Instruments X-Supreme 8000 with duplicate ICP-MS reference analysis. Fe is represented by *grey circle* (◯) and Zn with *black square* (■)

**Fig. 3:**
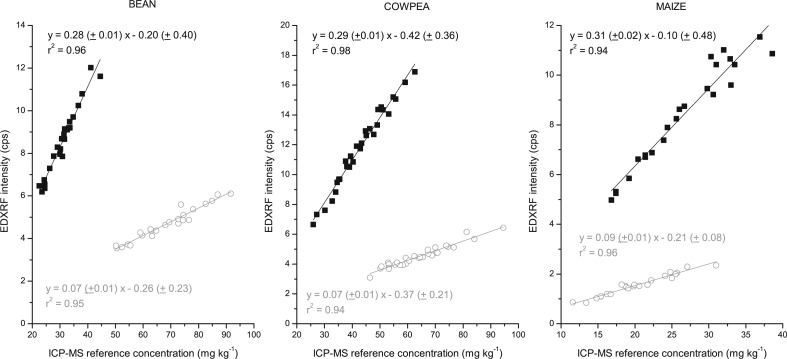
EDXRF calibration for bean, cowpea and maize flour with the Bruker S2 Ranger. Fe is represented by *grey circle* (◯) and Zn with *black square* (■)

The results for the bean, cowpea and maize flour calibration are shown below for both the Oxford and Bruker EDXRF instruments (Figs. 2 and 3 respectively). There was a strong correlation (r^2^ > 0.92) between the EDXRF intensity and the reference ICP-MS concentration for the flour samples in each of the crops tested with both instruments, as shown in Table 2 and Figs. 2 and 3. The standard error of calibration was <3 mg kg^–1^ for Fe and Zn in all the crops and both instruments.

**Table 2:** Calibration statistics for beans, cowpea and maize for Oxford Instruments and Bruker XRF instruments

Species	Analyte	Range (mg kg^–1^)	Mean (mg kg^–1^)	Sample no.	Oxford Instruments		Bruker	
					r^2^	SEC (mg kg^–1^)	r^2^	SEC (mg kg^–1^)
Bean	Fe	50.2–91.6	68.4	24	0.96	±2.4	0.96	± 2.8
	Zn	22.5–44.5	31.1	24	0.92	± 1.7	0.92	±1.1
Cowpea	Fe	46.4–94.5	65.4	25	0.94	± 2.6	0.94	± 2.9
	Zn	25.9–62.2	43.4	25	0.96	± 2.6	0.98	±1.5
Maize	Fe	11.6–31.0	20.2	23	0.90	±1.9	0.92	±1.5
	Zn	16.8–38.6	26.6	23	0.92	± 2.1	0.95	±1.5

### Limits of quantification

The limits of quantification are shown in Table 3, and confirm these values are comparable between the two instruments and more than suitable for high-throughput screening in breeding programs.

**Table 3:** Limit of quantification for Fe and Zn for each of the crops

Instrument	Crop	Fe (mg kg^–1^)	Zn (mg kg^–1^)
Oxford Instruments	Maize	9.8	8.8
	Beans	18.6	9.5
	Cowpea	18.5	9.7
Bruker	Maize	11.3	5.9
	Beans	16.3	8.5
	Cowpea	17.3	8.8

### Validation

**Fig. 4:**
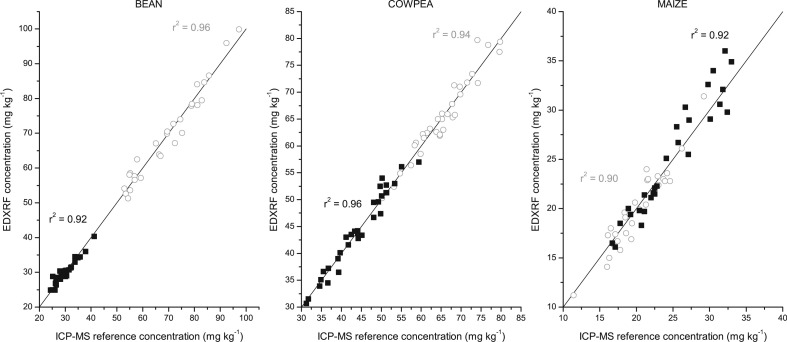
Correlation between reference ICP-MS analysis and EDXRF analysis for bean, cowpea and maize flour with the Oxford Instruments X-Supreme 8000. Fe is represented by *grey circle* (◯) and Zn with *black square* (■) with y = x represented by the solid line

**Fig. 5:**
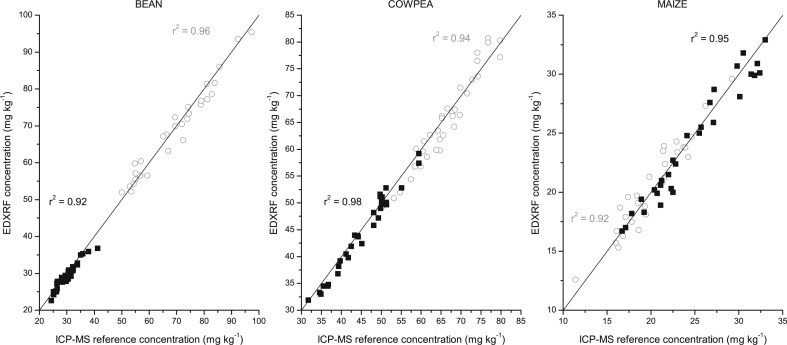
Correlation between reference ICP-MS analysis and EDXRF analysis for bean, cowpea and maize flour with the Bruker S2 Ranger. Fe is represented by *grey circle* (◯) and Zn with *black square* (■) with y = x represented by the solid line

To validate the calibration methods, a set of 28 samples for each of the crops with a range of Fe and Zn concentrations were analysed with the crop specific XRF calibration method. When comparing the XRF results with the reference (duplicate) ICP-MS analysis, there was a strong (r^2^ > 0.9) correlation for both Fe and Zn in each crop, (Fig. 4 and Fig. 5 for Oxford Instruments and Bruker XRFs, respectively). As detailed in Table 4, the average bias between the XRF and ICP-MS results was less than 1 mg kg and not significantly different from zero (per the paired t-tests). The calculated confidence interval indicates that the XRF results could be expected to be within arange of ± 2.3 and ± 5.5 mg kg^–1^ (an average of ± 3.3 mg kg^–1^) between the XRF results and that of the reference ICP-MS analysis across the 3 crops and 2 elements with both instruments (detailed in Table 4).

**Table 4:** Validation statistics

Method	Statistic	Bean		Cowpea		Maize	
		Fe	Zn	Fe	Zn	Fe	Zn
ICP-OES	Range	50.0–83.8	24.3–37.9	50.2–79.7	28.5–59.4	11.4–30.1	16.7–37.6
	Avg RSD	0.8	0.7	0.9	0.9	1.7	1.5
Oxford Instruments	r^2^	0.97	0.93	0.97	0.98	0.91	0.92
X-Supreme 8000	SEP	±2.8	±1.7	±1.8	±1.4	±1.3	±1.8
	95% CI	±5.5	± 3.3	±3.5	± 2.8	±2.5	±3.5
	Bias^a^	- 0.18	+ 0.36	- 0.05	- 0.08	+ 0.13	- 0.34
	Avg SD	1.0	2.5	0.72	1.9	0.58	2.5
	Avg RSD	1.6	3.0	1.1	1.1	1.8	1.5
Bruker	r^2^	0.96	0.93	0.98	0.99	0.92	0.95
S2 Ranger	SEP	±2.5	±1.5	±1.9	±1.4	±1.2	±1.2
	95% CI	±5.0	± 3.0	± 3.7	± 2.8	±2.3	±2.3
	Bias^a^	+ 0.46	+ 0.93	+ 0.60	+ 0.81	- 0.37	+ 0.50
	Avg SD	1.3	0.49	1.5	0.52	0.77	0.44
	Avg RSD	2.0	1.7	2.3	1.2	3.8	1.9

All units are presented as mg kg^-1^ , apart from RSD (%) and r^2^

The reproducibility between XRF analyses was determined from the analyses of validation samples in duplicate for each crop. The variability was <4% between replicates across the study.

## Discussion

Results presented in this study indicate that direct whole grain analysis by XRF is not feasible for screening grains larger than wheat. We have shown that with single XRF analysis of large grains, the correlation between the XRF intensity and the ICP reference analysis is weak (r^2^ = 0.33 and 0.65 for Fe and Zn respectively) and is only improved marginally when triplicate analyses are averaged (r^2^ = 0.47 and 0.81). Furthermore, the reproducibility between replicates is poor (max COV > 12% for both Fe and Zn) when compared to that found when analysing smaller whole grains with XRF (Paltridge et al. 2012a, b). The Fe calibration is particularly poor, and as this is a target element for bean crops, this further demonstrates that whole grain XRF analysis will not be suitable. This is not surprising considering the size of the grain studied here, which has an average diameter double that of the crops analysed in previous reports (shown in Table 5). An increase in seed diameter will result in a decrease in the number of grains incident to the x-ray radiation “window” and a poor representative sub-sample analysed. Furthermore, the geometry of the larger grains are more spherical than that of the large diameter small grains (wheat and rice, Table 5) and this will further reduce the packing density and increase the air cross-section in the analysed samples of these larger seeds (Donev et al. 2004; Weitz 2004; Li et al. 2010). The results of these packing effects are likely to be some of the possible causes of the poor correlation between XRF response and ICP-MS analysis shown in Fig. 1.

**Table 5:** Physical properties of grains analysed with XRF within HarvestPlus program

	Crop	Diameter (mm)	Sphericity (%)	Reference
Small grains	Pearl Millet	2^a^	≈ 94	(Jain 1997)
	Rice	3^b^	≈ 40	(Varnamkhasti et al. 2008)
	Wheat	4^c^	≈ 55	(Gürsoy and Güzel 2010)
Large grains	Maize	6^c^	≈ 70	(Karababa 2006)
	Bean	8^c^	≈ 62	(Kara et al. 2013)
	Cowpea	7^c^	≈ 77	(Kabas et al. 2007)

a mean diameter

b equivalent diameter

c geometric mean diameter

Grinding the grain to flour significantly improved both the calibration statistics (r^2^ ≥ 0.93) and the reproducibility between replicates (max COV = 5.3 and 3.5% for Fe and Zn). There are some associated disadvantages to analysing flour samples rather than whole grain. These include: increased sample preparation leading to possible reduction in throughput; increased risk of contamination during sample grinding; increased cost per analysis as the sample cups need to be re-made between each XRF analysis and the additional labour costs required to grind samples. Despite these added steps the analysis is still faster and cheaper than the standard AAS and ICP analyses, which also require samples to be ground prior to analysis (Pfeiffer and McClafferty 2007a). Consequently, it is concluded that screening these crops with XRF is still a useful alternative to conventional micronutrient analyses. The validation indicates that the results from the XRF can be expected to be within ±5.5 mg kg^–1^ of the ICP-MS results for Fe and within ±3.5 mg kg^–1^ for Zn, with an average bias of <1 mg kg^–1^ with the XRF results in comparison to the ICP-MS results.

We have previously proposed the use of a single calibration method for analysing multiple crops, but this has been deemed unsuitable for previous whole grain analyses due to the significant matrix effect observed when analysing whole grain crops (Paltridge et al. 2012b). It was anticipated that with flour analysis, this matrix effect may have been mitigated, thereby enabling a single calibration method to analyse all these larger grain crops. Unfortunately, from the data shown here it is evident that the calibration equations are not similar enough to enable this without compromising accuracy for XRF screening.

**Fig. 6:**
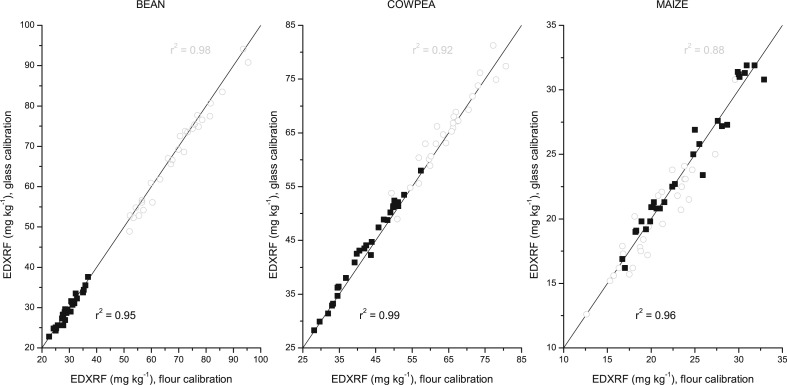
Validation of bean, cowpea and maize with non-matrix matched (glass) calibration and respective flour calibration. Fe represented as *grey circle* (◯), Zn shown as *black square* (■) and respective validation statistics shown in the respective colour with y = x represented by the solid line

We have previously shown the benefits of employing a non-matrix matched calibration for calibrating XRF instruments for the analysis of wheat, rice and pearl millet (Guild and Stangoulis 2016). Using glass beads to calibrate the instrument this eliminates the need to carry a plant material internationally when calibrating a new instrument, hereby reducing quarantine difficulties. We have employed this same approach for the crops studied here. The glass calibration was recently developed (Guild and Stangoulis 2016) with 10 glasses each analysed with the appropriate flour calibration method five times, and the average of these results averaged to determine a “matrix adjusted” calibration set. The analysis of the validation set with the developed glass calibration correlates strongly with the flour calibration XRF analysis (Fig. 6). The correlation between the two calibration methods is robust for all crops and elements. The correlation for Fe in maize was the weakest (r^2^ ≥ 0.88) and is likely due to the low range in Fe concentrations in both the calibration and validation sets. In addition, the response for Fe with EDXRF was lower than that for Zn, which is likely to compound the slightly lower accuracy observed with Fe in this crop. Despite this slightly weaker correlation between the two calibration methods, this still demonstrates the suitability of this method as Fe is not a target micronutrient in the HarvestPlus Maize program. These results demonstrate that employing a non-matrix matched calibration is still ideal for establishing a suitably robust calibration on instruments across different breeding laboratories. Furthermore, the added benefit of not having to carry plant material internationally and the other associated problems encountered when using plant material for instrument calibrations demonstrates the suitability of this approach.

## Conclusions

We have demonstrated the application of EDXRF for high-throughput screening of Fe and Zn concentration in common bean, maize and cowpea, in both Oxford Instruments X-Supreme 8000 and the Bruker S2 Ranger. The larger grain size of these crops, when compared to those previously studied (ie wheat, rice and pearl millet), results in a higher variability when analysing whole grain. Consequently, samples must be ground to flour prior to analysis with EDXRF to ensure suitable accuracy. As discussed by Paltridge et al. (2012a, b), the high throughput of EDXRF make this technique highly suitable for application in biofortification breeding programs. The added benefits of reduced operating costs with minimal consumables and minimal sample preparation along with the ease of operation and minimal laboratory requirements makes this technique ideal for screening crops on site without requiring specialised laboratories and highly specialised staff. The throughput of micronutrient screening with this technique has the potential to largely replace other methods for the measurement of these elements, hence saving both time and economic resources. We have now reported the use of EDXRF for screening Fe and Zn in wheat, rice, pearl millet, maize, beans and cowpea which encompasses all the primary crops targeted for Fe and Zn breeding in HarvestPlus to date. This technique has already been well utilised across biofortification breeding programs at international research centres of the CGIAR (Consultative Group for International Agricultural Research) and NARS (National Agriculture Research Systems) with tens of thousands of samples analysed in the past few years.
